# A survey of resident physicians’ perceptions of competency-based education in standardized resident training in China: a preliminary study

**DOI:** 10.1186/s12909-022-03863-0

**Published:** 2022-11-17

**Authors:** Qi Chen, Ming Li, Na Wu, Xue Peng, GuangMin Tang, Heng Cheng, LiuLing Hu, Bin Yang, ZhongLi Liao

**Affiliations:** 1 Department of Anesthesiology, Chongqing University Cancer Hospital, 400030 Chongqing, PR China; 2 Health management center, the First Affiliated Hospital, Army Military Medical University, 400038 Chongqing, PR China; 3 Department of Epidemiology, College of Preventive Medicine, Army Medical University, 400038 Chongqing, PR China; 4 Department of Gastroenterology, the Second Affiliated Hospital, Army Military Medical University, 400037 Chongqing, PR China; 5 Center of Infectious diseases, West China Hospital, 610041 ChenDu, PR China; 6 Department of Gastroenterology, the First Affiliated Hospital, Medical and Pharmaceutical College, 401331 Chongqing, PR China; 7 Department of Gastroenterology, Chongqing University Cancer Hospital, 400030 Chongqing, PR China; 8 Department of Anesthesiology, the First Affiliated Hospital, XiaMen University, 361003 FuJian, PR China

**Keywords:** Competency-based medical education, Resident physicians, Standardized resident training

## Abstract

**Background:**

Understanding resident physicians’ perceptions of competency-based medical education(CBME) may help improve approaches for implementing such education in standardized resident training (SRT). We conducted surveys of residents in China to identify their perceptions of CBME and determine the degree to which such education impacts their career plans.

**Methods:**

Questionnaire contained a total of 24 questions, which were answered using multiple choice or yes/no, was distributed to residents who were undergoing SRT, regardless of specialty, at 7 accredited training bases located across six provinces of China. The survey aimed to investigate residents’ reasons for participating in SRT, perceptions of CBME, interest in receiving CBME-associated courses, and attitudes towards CBME.

**Results:**

Overall, 441 residents completed the questionnaire.17.7% (78/441) responded “no clear objective” before the participated in SRT. Only 3.9% (17/441) fully understood the objectives, training contents, and assessment system of the current “competency-based” standardized training program for residents in China. Residents ranked clinical skills and patient care, interpersonal communication, and professionalism, as the three most important competencies. Most were interested in the CBME residency programs. 90.7% felt that implementing CBME could help them clarify their professional direction and improve their career planning.

**Conclusion:**

Residents had positive perceptions of the incorporation of CBME into SRT. Administrators, educational leaders, and clinical faculty should seek to further publicize and increase the popularity of CBME.

**Supplementary Information:**

The online version contains supplementary material available at 10.1186/s12909-022-03863-0.

## Background

Training medical residents is internationally recognized as a continuing education process that aims to cultivate novice physicians’ ability to work independently. China’s resident-training program has undergone important changes in recent decades. In the 1990s, the objective of the program was to provide continuing education that transformed medical graduates into qualified clinical physicians; however, a prominent characteristic of this period was that resident training varied considerably across different hospitals. Each province conducted its training according to its own standards. In order to standardise the quality of Chinese doctor, standardized resident training (SRT) mainly led by the National Health and Family Planning Commission (NHFPC) was launched in China in 2014. The Chinese Medical Doctor Association (CMDA) under direct governance of the NHFPC has been commissioned to manage the details of SRT (e.g., creating training content and accreditation standards).The SRT tracks residents into 36 specialties: 27 for clinical medicine, 7 for dentistry, and 2 for traditional Chinese medicine (TCM). The length of the US residency training or the UK specialty training varies according to the specialties.However, China’s SRT programs were set at 3 years in length regardless of specialty and residents had to comply with prescribed guidelines from Chinese national government issued curriculum document for each specialty in details [1]. The rotation process for each specialty consisted of three sections: ‘rotation purpose’(i.e. topics to understand or master), ‘basic requirements’(i.e. lists of diseases and skills and the number of cases required for the rotation), and ‘advanced requirements’. Medical graduates in China, after completing schooling, are required to finish 33 months of training in different departments in their chosen specialties and finally pass the residency certification exam. Since 2020, the adoption of SRT in a standardized pattern as mentioned above has been mandated across the country [2]. From this angle, SRT in China is an essential means through which medical graduates receive training in the sub-specialty of their chosen career. Residents can only be employed in hospitals after successful completion of this program. Thus, the SRT has an important influence on China’s overall health-care system and medical services.

With the continuous world wide development of the 3rd generation of reformation in medical education, competency-based medical education (CBME) has become a global reform movement [3]. CBME is a redesigned framework for health-professional education that emphasizes the acquisition of demonstrable competencies throughout residency. This approach seeks to transform traditional time-based and teacher-centered learning into performance-based and learner-centered learning, and has been adopted in many countries (e.g., Canada, the United States, and the United Kingdom) [4–7]. When compared with previous postgraduate medical education, CBME comprises seven key fundamental characteristics [8]: 1) Its primary goal is for graduates to achieve their desired competencies; these competencies should be aligned with the roles the graduates will perform in the next stage of their careers. 2) The predefined competencies contained in CBME are derived from the needs of patients, learners, and institutions. 3) Time is considered a learning resource rather than the basis of progression of competence. 4) Teaching and learning experiences are sequenced to facilitate an explicitly defined, stage-based progression of learners’ ability. 5) Learning is tailored, in some manner, to each learner’s progression. 6) Numerous direct observations and focused feedback are used to assist effective learner development of expertise. 7) Assessment is planned, systematic, systemic, and integrative. These fundamental characteristics demonstrate a high focus on needs-based outcomes, authenticity, and learner-centeredness.

In alignment with the global medical-education community, the government-issued guidelines for SRT in China have been changing from focusing on process measures to emphasizing outcomes and competency [9]. In 2015, researchers from China Medical University have developed a competency framework that is consistent with the Chinese medical context [10]. It involves eight competency factors: information and management, professionalism, clinical skills and patient care, interpersonal communication, health promotion and disease prevention, medical knowledge, academic research skills, and teamwork.However, Chinese graduate medical education system lacks a mature competency framework to guide the education of residents for a long time. Some researchers and accredited training bases in China have begun to explore the construction of relevant curriculum systems based on the competency model. Implementation and evaluation of the curriculum are also discussed [11, 12]. Encouragingly, China Consortium of Elite Teaching Hospitals for Residency Education, a non-profit academic group composed of 9 leading teaching in China, including Peking Union Medical College Hospital, Peking University first hospital, Fudan University Zhongshan Hospital, Sichuan University West China Hospital, the first affiliated hospital of Zhejiang University school of medicine, Central South University Xiangya Hospital, the first affiliated hospital of Sun Yat-sen University, Hong Kong University Li Jiacheng Medical College (Queen Mary Hospital) and Peking University Third Hospital issued the first consensus on the core competency framework for resident doctors in China in 2018 [13], which marks the on-set for the transformation of the standardized training of Chinese residents to competency oriented medical education. The framework consists of six core competences:professionalism, medical knowledge and skill, patient care, communication and collaboration, teaching and life learning.The consensus provided an important theoretical basis for future exploration of reform in SRT such as setting training goal, curriculum design, assessment and evaluation, faculty development and teaching-resources improvement, etc. However, the scale of the training programs and the number of residencies is huge and the level of training quality is mal-distributed and imbalanced, so the implementation in the future will face great challenges and still needs to be constantly revised and improved in practice. Much of the literature concerning competency-based training is written from the perspective of educators and administrators who are involved in designing, planning, and overseeing the implementation of CBME. As we know, medical residents are the main participants in CBME, it is important to understand how they view CBME and their perceptions of CBME may help inform approaches for effectively incorporating CBME in SRT. SRT in China is at the stage of transition to CBME. There are few published studies that examine residents’ perspectives in China. Considering this, we conducted this preliminary survey to identify Chinese residents’ perceptions of CBME. We also sought to determine whether experience of CBME impacts such residents’ career decisions.

## Methods

Anonymous online questionnaire was distributed to medical residents located across six provinces in China (Chongqing, Sichuan, GuiZhou, Yunnan, JiangSu, and FuJian). The questionnaire contained a total of 24 questions, which were answered using multiple choice or yes/no. Detailed descriptions of the questionnaire are provided in Supplementary Material S1. The survey was divided into five sections: demographic characteristics, reason for participating in SRT, perception of CBME, interest in CBME-associated courses included in SRT, and attitude towards CBME.

The survey was administered online through the “wenjuanxing” platform (www.wjx.cn, China), and included an information letter that outlined the research goals and the voluntary and anonymous nature of the survey. A survey link was sent to all participants via WeChat, and reminders were sent at 7, 14, and 21 days. The survey was closed at 30 days after posting, after which responses could not be submitted. Data were collected from January 1, 2022, to January 31, 2022. This study was approved by the Ethics Committee of ChongQing University Cancer Hospital in ChongQing (registration number: CZLS20222068).

Questionnaires collected were included from analysis as follows criteria: respondents who were undergoing standardized training, regardless of specialty; Exclusive criteria: respondents who populated the answers mechanically (e.g., filled each question with identical answers); respondents who submitted multiple questionnaires using the same Internet Protocol (IP) address (in this case, the last questionnaire submitted would be treated as valid input, with the rest, discarded).

All variables were described using numbers (n) and percentages (%). All statistical procedures were performed using IBM SPSS Statistics for Windows, version 22.0 (IBM Corp., Armonk, NY, USA); Fisher exact tests were used to test group differences. *P*-values of less than 0.05 were considered to indicate statistical significance.

## Results

It is assumed that the questionnaire was distributed to about 480 residents and a total of 441 residents completed the questionnaires.The response rate is about 91.8%. The average age, proportion of women, and marital status are described in Table 1. Overall, 42% (185/441) of the respondents were men, and 58% (256/441) were women; 38.8% (171/441) were in their first year of SRT, 32.7% (126/441) were in their second year, and 28.6% (144/441) were in their third year; 71.4% (315/441) had a bachelor’s degree, 26.5% (117/441) had a master’s degree, and 2% (9/441) had a PhD. Before commencing SRT, 36.7% (162/441) had work experience, while 63.3%(279/441) did not have any work experience.

**Table 1:** Demographics of residents completing the survey

	N(%)
Gender	
Male	185(42)
Female	256(58)
Education	
Bachelor’s	315(71.4)
Master’s	117(26.5)
PhD	9(2)
Year of Training	
First	171(38.8)
Second	126(32.7)
Third	144(28.6)
Work experience	
Yes	162(36.7)
No	279(63.3)
Department	
Medicine	212(48.1)
Surgery	131(29.7)
Gynecology and Pediatrics	38(8.6)
Others	60(13.6)

## Residents’ reasons for participating in SRT

The respondents were asked a series of questions designed to gauge their reasons for participating in SRT (Table 2). Overall, 36.3% (160/441) indicated that they participated in SRT because of work-union requirements, 27.7% (122/441) chose “improve my professional title” and, surprisingly, 17.7% (78/441) responded “no clear objective”; this was especially common among respondents with no work experience. Only 3.9% (17/441) of the residents stated that they fully understood the objectives, training contents, and assessment system for the current “competency-based” standardized training program for resident physicians in China; 64.4% (284/441) responded “partially understand,” and 31.7% (140/441) responded “do not understand.” Of the total sample, 68.7% (303/441) reported that they had studied and understood the objectives, training contents, and assessment system of the standardized training program before they applied to participate. The top three means by which they obtained this information were: through networks and mobile terminals (52.8%), through special lectures on live and training bases (24.1%), and through discussions with experienced friends and classmates (17.8%), respectively. The result had some similarities with a survey from Canada, which residents received information from training bases or hearsay from friends and colleagues [14]. The main reasons the residents chose the training major were: career planning (42.6%), previous study and major (36.5%), and self-interest (13.2%), respectively.

**Table 2:** Residents’ purpose of participating in SRT

	The reason why you are attending the SRT program?N(%)
The requirement for applying for a job	160(36.3)
Professional title	122(27.7)
No clear objectives	78(17.7)
others	81(18.3)
	Do you know the objectives, training contents and assessment system of the current “competency-based” standardized training program for residents in China? N(%)
Don’t understand	140(31.7)
Partially understand	284(64.4)
Fully understand	17(3.9)
	Have you ever studied and understood the objectives, training contents and assessment system of CBME in SRT program for residents before you signed up for it? N(%)
Yes	303(68.7)
No	138(31.3)
	how did you obtain the relevant information? N(%)
Through the network and mobile terminals	160(52.8)
Experience introduction from friends and classmates	54(17.8)
Special lecture on live and training base	73(24.1)
Others	16(5.3)
	Is the training you actually participated in in line with your desired major? N(%)
Yes	386(87.5)
No	55(12.5)
	What is the basis of your choice of training major? N(%)
Career planning	188(42.6)
Previous study and major	161(36.5)
Self-interest	58(13.2)
Choose at will	20(4.5)
Others	14(3.2)

## Residents’ perception of CBME in SRT

**Fig. 1:**
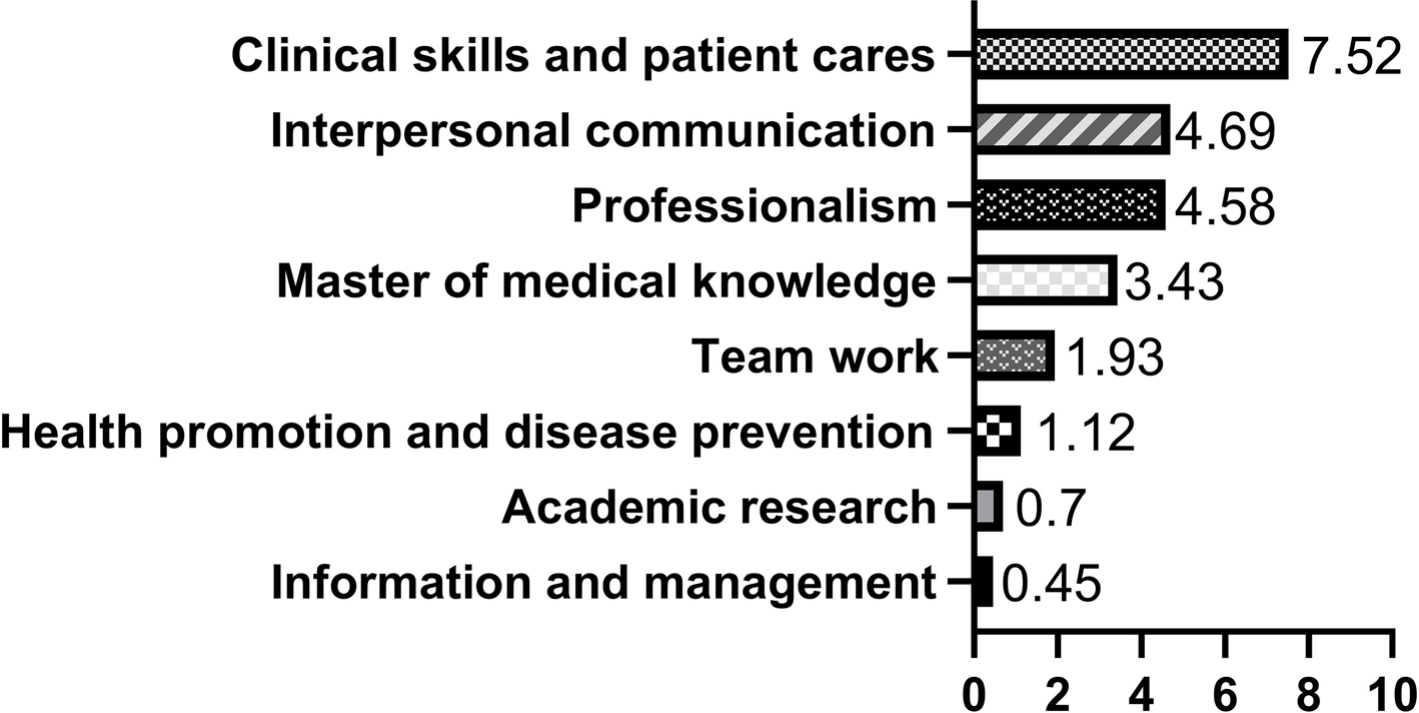
Score bar plot of ranking top 3 competences out of 8 options

To gauge residents’ understanding and perception of CBME, respondents were asked to list the three most important CBME competencies (in their own opinion); this was conducted using the question: In what areas do you think a clinician should have the most competence (please choose the three you think are most important)? It’s a ranking question. The CBME competency item was ranked by comprehensive average score. The first three items were assigned different score (1st rank score: 3, 2nd rank score: 2, 3rd rank score: 1). And then, the comprehensive average score was calculated according to the formula:Comprehensive average score = Weight * frequency / person-time responded to this question. The results were as follows: clinical skills and patient care (7.52), interpersonal communication (4.69), professionalism (4.58; see Fig. 1). In Canada, where CBME is well-established, referring to the concept of what residents were expected to know and to do, all three terms were used to express standards of clinical knowledge and performance [14]. Further more, they expressed an expectation that CBME would provide greater latitude for self-directed learning.

## Residents’ interest in CBME-based courses in SRT

It is expected that, through engaging in CBME-based residency programs, residents gain more in-depth exposure to clinical practice and enjoy greater involvement in the entire process of patient management. Courses on medical humanities and interpersonal communication should also be offered in such programs. Most of the residents showed an interest in such CBME residency programs. Both residents with and without prior work experience were willing to participate in various scientific research and academic activities; there were no statistical differences between these two groups in this regard (*P* = 0.051). When they were asked “when do you think is the most appropriate time to conduct scientific research and academic activities?,” 49.7% chose “special time periods should be specifically devoted to research activities.” All of these responses are summarized in Table 3.

**Table 3:** Interest of CBME in SRT

	In addition to the traditional professional theory teaching, the SRT program will also involve the contents of medical humanities and interpersonal communication. Are you interested in it?N(%)
Yes	415(94.1)
No	26(5.9)
	In the SRT, more time needs to be spent on patient whole-process management and various clinical practices. Are you interested in this? N(%)
Yes	425(96.4)
No	16(3.6)
	Are you interested in participating in scientific research and academic activities during your training? N(%)
Yes	361(81.9)
No	80(18.1)
	what time do you think is appropriate for scientific research and academic activities N(%)
Time devoted to research activities	219(49.7)
Extra time in addition to routine training	150(34.0)
Others	72(16.3)

## Residents’ attitude towards CBME in SRT

Surprisingly, 26.5% of the respondents reported thinking of changing their training major during their training. The main reasons for this were “I have developed new interests during the training process” (42.7%) and “I am considering changing my career” (38.5%). Further, 20.4% of the respondents had thought of quitting the SRT. The main reasons here were “I am considering changing my career” (48.9%) and “I am unable to adapt to the requirements of SRT and the associated assessments” (22.2%). Overall, most (90.7%) of the respondents indicated that inclusion of CBME in SRT helped them clarify their professional direction and improve their career planning (Table 4). There were no statistical differences among different years of training in this regard (*P* = 0.056).

**Table 4:** Residents’ attitude towards CBME in SRT

	Have you ever thought of changing your training major during your training?N(%)
Yes	117(26.5)
No	324(73.5)
	the reason for changing your training major during your training N(%)
Develop new interests during training process	50(42.7)
Changing my career	45(38.5)
Others	22(18.8)
	Have you ever thought of quitting the SRT? N(%)
Yes	90(20.4)
No	351(79.6)
	the reason for quitting the SRT N(%)
Changing my career	44(48.9)
Unable to adapt to the requirements and and the associated assessments	20(22.2)
Others	26(28.9)
	Do you think inclusion of CBME in SRT help you clarify your professional direction and improve your career planning? N(%)
Yes	400(90.7)
No	41(9.3)

## Discussion

In 2014, the Chinese government, aiming at improving the quality of medical-resident graduates, released new standards for the resident training curriculum. In alignment with the global medical-education community, the first consensus on the core competency framework for resident doctors was issued in China in 2018, the focus of SRT is moving from a Flexner-type era to a competency-based era [15]. CBME features a new method of assessment, facilitates progression through residency, and provides opportunities for developing competency. Implementation of CBME represents a social construction process within a local and cultural context [16]. This study aimed to evaluate resident physicians’ understanding of CBME in China and how the implementation of CBME in a SRT program may affect their decisions to pursue this specialty. Over 90.7% of the residents in this survey believed that the implementation of CBME in an SRT program could help them clarify their professional direction and improve their career planning. Therefore, the training system could have a positive impact on career planning. However, only 3.9% of the residents felt that they fully understood the objectives, training contents, and assessment system of the current “competency-based” standardized resident training program in China. Moreover, most residents reported that their information regarding this training was not obtained from the government or official institutions. Most obtained information on SRT through networks or from friends, which may have affected their cognition of the training; there is also the risk that they did not obtain full details of the program, or that they received incorrect information. There are some similarities with a survey from Canada, which residents received information from training bases or hearsay from friends and colleagues. Referring to the experience of Canada, in order to combat uncertainties about what exactly constituted competence, and how it could be defined and assessed, residents advocated for clear communication with their program directors prior to and during the implementation process.These results gave us an inspiration that improving understanding of the goals and rules of CBME may help residents to identify and address their own learning needs, and standardize themselves during the training process.Thus, it is necessary for administrators, educational leaders, and clinical faculty to take steps to further publicize and increase the popularity of CBME in SRT to ensure that residents could receive comprehensive and correct information before attending SRT. Administrators, educational leaders, and clinical faculty should also provide the residents with some career advice and assessment of their work skills and abilities to successfully complete a residency, which will help the residents to set a plan for improvement before participating in SRT and give them a better chance at being matched to competency-based SRT.The specific and detailed requirements for competency based training also need to be made more explicit in the future.

China’s health system and medical culture are very different from those in the West [17]. These different professional demands mean that the practical work experience of residents in China is very different from that of residents in the West. Unlike other countries, the composition of the resident population in China includes local resident trainees employed by hospitals (e.g., township, county, prefectural, provincial, and national hospitals) and clinical postgraduates of medical universities. Differences in terms of background, age, medical school, abilities, work efficiency, level of income, and future career goals may cause residents to have different perceptions and demands of CBME during their training. Especially, some residency trainees in China are permanent employees who will work in the same hospital virtually from their entry into an SRT position until they retire. Job permanence and poor mobility jeopardise incentives may be an obstacle for practitioners to seek quality standardization. However, the results of this survey showed that most residents felt competencies relating to other broader aspects of professionalism, communication and teamwork, besides clinical skills, were important, and they showed their interests in the inclusion of associated courses in their CBME programs. In 2016, a survey was carried out to understand residents’ perceptions of their training programs at a teaching hospital in China [18]. The results demonstrated residents were unable to differentiate well between different competency categories,since it lacked a mature competency framework at that time. However, when it comes to the perception of program organization,residents noted case discussions and journal clubs were less frequent, which may provide an environment conducive to medical knowledge learning. Compared with these residents in Lio’s study, residents participated in our study expected to gain more in-depth exposure to clinical practice and enjoy greater involvement in the entire process of patient management, which is accord with the current requirements of CBME-based SRT. Given the high doctor–patient tensions in China, educators and administrators should try to explore more effective ways to help residents participating in patient management extensively. China is at the stage of transition to to CBME. The next step forward should focus on further improvement of establishing detailed CBME program. China has launched the key steps toward a national SRT system to produce doctors with the professional, clinical, ethical, and human competencies. It will take a long time, perhaps decades, to achieve uniform quality standard across all of China, which required concerted efforts of all parties, not only educators and administrators, but also resident physicians themselves.

Several limitations of this study should be considered. Firstly, the main limitation of the study is the representativeness of the data. In order to ensure that our participants represented as diverse a population as possible, we included residents from a variety of specialties and years of training. However, the scale of the training programs and the number of residencies is huge and the level of training quality is mal-distributed and imbalanced in China. There are accredited 559 training bases geographically distributed across China. The capability of clinical teaching faculty is mixed. To ensure the nation wide implementation of the CBME-based SRT, further study for residents from different grade of hospitals is necessary. Secondly, an additional assessment to check residents’ understanding of CBME should be done, ensuring an adequate level of baseline knowledge, because the validity of these responses may be compromised by pre-existing misconceptions about CBME.Finally, our survey was carried out via questionnaire only contained a total of 24 questions, which were answered using multiple choice or yes/no. Further study should choose questionnaire survey and semi-structured interviews as the research method to understand the complexities of residents’ perceptions.

## Conclusion

This study demonstrated that the residents analyzed in this survey had positive perceptions of CBME being incorporated into SRT in China. This feedback regarding residents’ interest in and perceptions and demands of CBME in SRT may provide a theoretical basis for administrators, educational leaders, and clinical faculty members to establish a framework consistent with the Chinese medical context.

## Supplementary Information

**Additional file 1.**

## Data Availability

The original contributions presented in the study are included in the article, further inquiries can be directed to the corresponding author.

## Abbreviations

SRT: standardized resident training
NHFPC: National Health and Family Planning Commission
CMDA: Chinese Medical Doctor Association
CBME: competency-based medical education
