# Supplementary material for: A survey of resident physicians’ perceptions of competency-based education in standardized resident training in China: a preliminary study
Source: BMC Med Educ. 2022 Nov 17;22:801. doi: 10.1186/s12909-022-03863-0 (PMC9673373; doi:10.1186/s12909-022-03863-0)
Supplement: Supplementary file 1 — Additional file 1. [file 12909_2022_3863_MOESM1_ESM.docx]

**A survey of resident physicians’ perceptions of competency-based education in standardized resident training in China: A cross-sectional study**

1、Gender

(1)man

(2)woman

2、You are currently working in your province or city

3、What is your degree?

(1) Bachelor

(2) Master

(3) Ph.D

4、What was your major in medical school?

(1) Clinical Medicine (not classified)

(2) Internal medicine

(3) Surgery

(4) Department of Gynecology

(5) Pediatrics

(6) Department of Stomatology

(7) Department of Anesthesiology

(8) Emergency Department

(9) Department of Medical Imaging

(10) Nuclear medicine

(11) Clinical Pathology Department

(12) Laboratory Department

(13) General Practice

(14) Department of Oncology

(15) Others

5、Did you have any work experiences before commencing SRT?

(1)yes

(2) no

6、Time spent in clinical practice before commencing SRT.

(1) no

(2) ≤1 month

(3) ≤3 months

(4) ≤ half a year

(5) ≤1 year

(6) > 1 year

7、What grade are you in now?

(1) First grade

(2) Second grade

(3) Third grade

8、The reason why you are attending the standardized residency program?

(1) Work-union requirements

(2) Improve my professional title

(3) No clear objectives

(4) Others

9、Have you ever studied and understood the objectives, training contents and assessment of SRT before you signed up for it?

(1)yes

(2) no

10、If the answer to the previous question was "Yes", how did you obtain the relevant information?

(1) Networks and mobile terminals

(2) Discussions with experienced friends and classmates

(3)Special lectures on live and training bases

(4) Others

11、 Do you know the objectives, training contents and assessment system of the current "competency-oriented" standardized training plan for resident physicians in China?

(1) Don't understand

(2) Partially understand

(3) Fully understand

12、What is your major in SRT?

(1) Internal medicine

(2) Surgery

(3) Department of Gynecology

(4) Pediatrics

(5) Department of Stomatology

(6) Anesthesiology Department

(7) Emergency Department

(8) Department of Medical Imaging

(9) Nuclear medicine

(10) Clinical Pathology Department

(11) Laboratory Department

(12) General Practice

(13) Department of Oncology

(14) Others

13、Is the training you actually participated in in line with your desired major?

(1)yes

(2) no

14、What is the basis of your choice of training major?

(1) Career planning

(2) Self-interest

(3) Previous study and major

(4) Others

15、what do you think a clinician should have the post competence :(please choose the three you think are most important)

(1) Clinical skills and and patient care

(2) professionalism

(3) interpersonal communication

(4) Team work

(5) Health promotion and disease prevention

(6) Master of medical knowledge

(7) Information and management

(8) Academic research

16、In addition to the traditional professional theory teaching, the standardized training for residents will also involve the contents of medical humanities and interpersonal communication. Are you interested in it?

(1)yes

(2) no

17、In the standardized training of residents, more time needs to be spent on patient whole-process management and various clinical practices. Are you interested in this?

(1)yes

(2) no

18、 Are you interested in participating in scientific research and academic activities during your stay?

(1)yes

(2) no

19、If you answered "yes" to the last question, when do you think is the most appropriate time to conduct scientific research and academic activities?

(1) Special time devoted to research activities

(2) Extra time in addition to routine training

(3) Others___

20、 Have you ever thought of changing your training major during your training?

(1)yes

(2) no

21、If the last answer is "yes", what do you think is the reason?

(1) Develop new interests during the training process

(2) Changing my career

(3) Others

22、 Have you ever thought of quitting the standardized training for residents?

(1)yes

(2) no

23、If the last answer is "yes", what do you think is the reason?

(1) Changing my career

(2) Unable to adapt to the SRT requirements and the associated assessments

(3) Others

24、Do you think inclusion of CBME in SRT help you clarify your professional direction and improve your career planning?

(1)yes

(2) no
